# Setting up a clinical trial in care homes: challenges encountered and recommendations for future research practice

**DOI:** 10.1186/s13104-015-1276-8

**Published:** 2015-07-16

**Authors:** Victoria Shepherd, Jacqui Nuttall, Kerenza Hood, Christopher C Butler

**Affiliations:** South East Wales Trials Unit, School of Medicine, Cardiff University, 7th Floor Neuadd Meirionnydd, Heath Park, Cardiff, CF14 4YS UK; Nuffield Department of Primary Care Health Sciences, University of Oxford, New Radcliffe House, Radcliffe Observatory Quarter, Woodstock Road, Oxford, OX2 6NW UK; Cochrane Institute of Primary Care and Public Health, School of Medicine, Cardiff University, 5th Floor Neuadd Meirionnydd, Heath Park, Cardiff, CF14 4YS UK

**Keywords:** Care homes, Older people, Clinical trial, Recruitment, Ethics, Low-risk intervention

## Abstract

**Background:**

Older adults in care homes have increasingly complex health care needs, and care provision should be evidence-based whenever possible. However, recruitment of frail, older people to research is a complex process and often results in care home residents being excluded from research participation. This paper draws on the experience of setting up a randomised controlled trial to determine the effectiveness of probiotics on antibiotic-associated diarrhoea in care home residents [Probiotics for Antibiotic Associated Diarrhoea in Care Homes (PAAD) Study] in Wales.

**Findings:**

Significant challenges were encountered setting up a clinical trial in care homes. There were a number of barriers and facilitative factors encountered that were unique to this research setting. The classification of the study intervention (a widely available food supplement with a low risk safety profile) as an investigational medicinal product, with the associated requirements including obtaining statutory approvals and research governance, had a major impact.

**Conclusion:**

The process for setting up a clinical trial of an investigational medicinal product in care homes has been more complex and time consuming than the process for setting up an observational study in the same setting, and clinical trials in other health care settings. We recommend regulatory changes to ensure approvals processes are more proportionate to risk and context, to ensure that care home residents have the opportunity to participate in research and are able to help generate much needed evidence to underpin care. Recommendations made may inform future research practice.

Trial registration: ISRCTN 25324586.

## Background

An ageing population has resulted in a rise in the number of people requiring long term care [1] and those in long term care having greater complex health care needs [2]. The need for the development of a more structured and evidence-based approach to care provision within care homes has been recognised [3]. However, recruitment of frail, older people to research is a complex process [4]. Development of strategies and infrastructures to conduct research in older populations in care homes has been recommended [5]. These include the creation of ‘research ready’ care homes [National Institute Health Research Enabling Research in Care Homes (NIHR ENRICH) programme] and guidance for all stakeholders on good research practice in care home settings [6].

This paper draws on the experience of setting up a randomised controlled trial (RCT) to determine the effectiveness of probiotics on antibiotic-associated diarrhoea in care home residents [Probiotics for Antibiotic Associated Diarrhoea in Care Homes (PAAD) Study]. The aim was to randomise 400 residents (200 per arm) from approximately 24 care homes in South Wales, commencing 2013. We aimed to implement this trial after an observational study of antibiotic use and associated diarrhoea in the same setting [7]. Although we did not implement the trial because of new emerging data, we encountered many specific unique challenges in setting up a clinical trial of an investigational medicinal product (CTIMP) in this largely research naïve environment that resulted in significant challenges and delay. Our experiences may inform research planning in the care home environment.

## Findings

### Clinical trial approval

The planned intervention was a probiotic preparation (VSL#3) that is commercially available to the general public as a food supplement. However, the Medicines and Healthcare products Regulatory Agency confirmed that its use in this trial was classified as a CTIMP and therefore required clinical trial approval. The classification of a pragmatic study involving a widely available food supplement with a low risk safety profile as a CTIMP had a significant impact. The disparity between the statutory authorisations required for an IMP, and the largely absent relevant documentation for a product generally characterised as a food supplement, resulted in significant delays in conducting the trial.

### Recruitment of care homes

Recruitment of care homes as sites was a challenging and lengthy process. Care homes can be regarded as ‘communities of care’ where managers act as ‘gatekeepers’ both in terms of access to residents (e.g. to invite participation in research) and access to health care, and can act as a barrier or a facilitator. The degree of autonomy will vary greatly in each care home, and the background of the manager will also differ greatly (clinical or non-clinical) and experiences of health care or research activity. Care home managers were generally supportive about conducting research, but required assurances that support would be provided by the research team, and that the additional workload for care home staff would be minimised. It was planned to embed research nurses in participating care homes to assist with study related activities.

The manager then sought agreement from the wider community of staff within the care home before agreeing to participate. Consent in care homes has been described as a two tiered process, where obtaining consent at institutional/community level is required before progressing to consent from individuals [8], described as ‘gaining entree’ [9]. The process for recruiting 24 care homes was originally expected to take 5 months, this was extended to 13 months due to the difficulties outlined. Strategies to support the delivery of research in this health care setting in England and Scotland, in particular the NIHR ENRICH programme supported by local clinical research networks (NIHR LCRN), do not currently extend to Wales.

### Identification of principal investigators

It is a regulatory requirement in the UK that each research site has a principal investigator (PI) responsible for the conduct of the trial at the site [10]. Whilst the care home manager (if a Registered Nurse) could act as PI, the decision whether a subject is eligible for entry into a CTIMP is considered to be a medical decision and, therefore, must be made by a medically qualified doctor in accordance with the regulations [10]. The care home resident’s general practitioner (GP) would be responsible for assessing eligibility and prescribing the IMP, which required the recruitment of GP practices as research sites in addition to the care homes themselves.

### GP involvement

Responsible clinicians are required to confirm that subjects are eligible to participate in a CTIMP, but not in an evaluation of a food supplement. As this was considered a CTIMP, GPs were required to agree that each care home resident who provided consent could participate. Several GPs from multiple practices often provide care in a single care home. The practical difficulties experienced when seeking all relevant GPs’ agreement to participate, and the requirements for each practice to be a trial site in terms of approvals, good clinical practice (GCP) training and study-specific training for each GP, proved to be a major barrier to conducting the trial. Strategies were developed to minimise the burden on GPs and to encourage their involvement. This included the availability of online GCP training along with drawing up a letter agreement in place of a more comprehensive practice agreement or formal contract.

### Ethics approval

Adults lacking capacity may only be included in a CTIMP if; (a) similar results are unlikely to be obtained with individuals who can provide informed consent for themselves; (b) the benefits of administering the medicinal product are expected to outweigh risk to the participant, and (c) the trial relates directly to a life-threatening or debilitating condition from which the subject suffers [10]. Data obtained from the preceding observational study showed that the majority of residents (71%) lacked capacity and that those without capacity were significantly more likely to be prescribed antibiotics, be more frail, be at greater nutritional risk, and more often suffer from antibiotic-associated diarrhoea [7]. These data were provided to the Research Ethics Committee to support plans to include residents without capacity, as they are most at risk of the adverse consequences of antibiotic use and most likely to benefit from the study findings. Due to the continued request for justification of those lacking capacity, gaining ethical approval from the South East Wales Research Ethics Committee took 4 months. The observational study, which also involved adults who lacked capacity, received ethical approval in 1 month. However as a non-CTIMP it was governed by the Mental Capacity Act 2005.

### Research governance

Research governance in care homes is complex due to their status as non-NHS institutions and the contractual and financial arrangements of the resident as either self-funding, Local Authority funded or a mix of both [11] and they may also receive NHS-funded care. This is further complicated by whether the care provider is a commercial or non-commercial organisation. The lack of clarity regarding responsibility for research governance for the care homes involved was further clouded by the classification of the trial as a CTIMP and that, whilst care homes would be the main sites, GP practices would also be sites but are NHS organisations. The study also covered a number of health boards and local authorities that resulted in each of these organisations conducted their own approvals process. Resources are available which provide information and advice about conducting research in care homes [12], whilst research governance toolkits have been developed in other independent health and social care settings [13] which could be further developed for this setting.

### Indemnity

Issues arose around the indemnity requirements for care homes participating in research, particularly CTIMPs, as non-NHS sites. Research potentially exposes participants and researchers to risks of harm, and indemnity is required to protect against the effects of such risks [6]. Indemnity for research activity in the UK is provided by the Study Sponsor who would be liable for any non-negligent harm resulting from activity conducted in accordance with the Study Protocol. However, any negligence on behalf of the site (such as giving an incorrect dose) would not be covered by this indemnity and, although the likelihood of a claim arising out of the trial was small, it would have had to be covered by the site insurance. The NHS is vicariously liable through NHS Indemnity; however non-NHS researchers are obliged to make their own arrangements. A requirement for additional indemnity further impacted on the time required for setting up a CTIMP in the care home sector and may impact on care homes agreeing to participate. Indemnity costs should be included in funding applications for future research in care homes.

## Conclusion

The process for setting up a clinical trial in care homes has been more complex and time consuming than the process for setting up an observational study in the same setting, and clinical trials in other health care settings. Information and advice is available for research teams conducting research with the care home sector, however there is limited practical support available outside England and Scotland. Ethical concerns regarding the inclusion of older adults lacking capacity, combined with non-NHS research governance and the multiple layers of permissions and agreement required at each level of the process are challenging.

Strategies must be developed to streamline the approvals process and minimise the impact to ensure that an evidence-based approach to health care provision within care homes can be developed. Further consideration needs to be given in low risk CTIMP on regulatory interpretation, over and above monitoring, to ensure processes are more proportionate to risk and context. The incoming clinical trials regulation [14] may address some of these issues by recognising the importance of improving treatments for vulnerable groups such as frail or older adults and the introduction of ‘low-intervention clinical trials’. This may increase the amount of research involving this, and other, vulnerable populations that already experience health inequalities as a result of a lack of evidence-based health care. Unless the situation changes, care home residents will continue to be declined participation and potential benefit from trials of low risk interventions.

## Abbreviations

CTIMP: clinical trial of an investigational medicinal product
ENRICH: National Institute for Health Research: Enabling Research in Care Homes Programme
GCP: good clinical practice
GP: general practitioner
NIHR LCRN: National Institute for Health Research: Local Clinical Research Network
PAAD: probiotics for antibiotic associated diarrhoea
PI: principal investigator
RCT: randomised controlled trial
